# Comment on “Identification of Differentially Expressed Genes in Pituitary Adenomas by Integrating Analysis of Microarray Data”

**DOI:** 10.1155/2017/2023530

**Published:** 2017-10-25

**Authors:** Mateusz Bujko

**Affiliations:** Department of Molecular and Translational Oncology, Maria Sklodowska-Curie Memorial Cancer Center and Institute of Oncology, Warsaw, Poland

I am working on the role of aberrant gene expression in the pathogenesis of nonfunctioning pituitary adenomas. I started my work with a search of the literature and deposited data from previous expression profiling experiments. Therefore, I have read the article “Identification of Differentially Expressed Genes in Pituitary Adenomas by Integrating Analysis of Microarray Data” published by Zhao et al. with great interest [1]. The authors performed a comprehensive analysis of gene expression in pituitary adenomas and normal pituitary, exploiting the data from five different microarray experiments, including different sample types. This is the only integrative analysis in pituitary adenomas that has been published to date.

This kind of study is essential to investigate molecular pathogenesis of pituitary adenomas as only a few microarray-based expression profiling studies were performed on samples of gland affected by this disease. Most of these investigations included a small number of patients, usually involving very few or no control samples from normal human pituitary tissue. Zhao et al. combined existing repositories of microarray data from three studies to collect expression profiles of 12 normal pituitary samples. According to the published methodology, six of these control samples are from the study reported by Hussaini et al. [2]. The respective data were deposited in the Gene Expression Omnibus (GEO) database under accession number GSE4237. I carefully checked the description of the samples from the five datasets that were analyzed by Zhao et al. [1] including those from GSE4237. In this dataset, four samples are clearly described as pituitary adenomas, while the remaining six (GEO accession numbers: GSM96624, GSM96625, GSM96626, GSM96627, GSM96628, and GSM96629) are described as “brain, pituitary,” and it appears that these six samples were treated as normal pituitary tissues by Zhao et al. and used as controls for integrative analysis. However, the available detailed description of these samples clearly indicates that they are not derived from normal pituitary but from resected pituitary tumors (four samples) and HP75 cell line (two samples). Accordingly, in the original article by Hussaini et al. published in the year 2007 [2] that describes the analysis of the data from this particular experiment, no normal pituitary controls were used. This means that the data from expression profiling of tumor tissue samples and cell lines were mistakenly used as normal controls for the integrative analysis performed by Zhao et al. [1]. Thus, six out of 12 samples were improperly included in the control group.

The aim of the research of Zhao et al. [1] was the identification of the genes that are differentially expressed in normal pituitary and pituitary adenomas. Obviously, the incorrect assignment of the samples affects the results of the comparisons, and thus, the conclusions from the whole analysis may be incorrect.

The integrative analysis of gene expression profiles in pituitary adenomas, based on the methodology used by Zhao et al., is still needed, and further research is expected to shed light on the pathogenesis of pituitary adenoma. Due to the fact that previously published large-scale expression analyses of pituitary adenomas included only a very small number of samples from normal pituitary, more subtle differences between normal and tumor tissue in the pituitary at the molecular level remain elusive. However, accurate selection of data is crucial for reliable outcome and its interpretation. For this reason, I feel obliged to point out the mistake that occurred in the analysis by Zhao et al. [1] and to encourage the authors to reanalyze the data.

## References

[1] Zhao P., Hu W., Wang H. (2015). Identification of differentially expressed genes in pituitary adenomas by integrating analysis of microarray data. *International Journal of Endocrinology*.

[2] Hussaini I. M., Trotter C., Zhao Y. (2007). Matrix metalloproteinase-9 is differentially expressed in nonfunctioning invasive and noninvasive pituitary adenomas and increases invasion in human pituitary adenoma cell line. *American Journal of Pathology*.

